# Rates and temporal onset of mental health disorders during inpatient rehabilitation after acute physical injury or illness: An observational cohort study

**DOI:** 10.1371/journal.pone.0338207

**Published:** 2025-12-31

**Authors:** Anja Schanke Sundet, Marianne Løvstad, Ingrid Amalia Havnes, Nada Andelic, Caroline Ustvedt, Grethe Månum, Helene Høye, Laila Skogstad, Cathrine Brunborg, Charles Henry Bombardier, Jennie Ponsford, Solveig Lægreid Hauger

**Affiliations:** 1 Department of Research and Education, Sunnaas Rehabilitation Hospital, Bjørnemyr, Norway; 2 Department of psychology, University of Oslo, Oslo, Norway; 3 Division of Mental Health and Addiction, Oslo University Hospital, Oslo, Norway; 4 Institute of Clinical Medicine, Faculty of Medicine, University of Oslo, Oslo, Norway; 5 Department of Physical Medicine and Rehabilitation, Oslo University Hospital, Oslo, Norway; 6 Center for Habilitation and Rehabilitation Models and Services (CHARM), Faculty of Medicine, Institute of Health and Society, University of Oslo, Oslo, Norway; 7 Department of Traumatic Brain Injury, Sunnaas Rehabilitation Hospital, Bjørnemyr, Norway; 8 Beitostølen Healthsports Centre, Beitostølen, Norway; 9 Varden Specialist Centre, Bjørnemyr, Norway; 10 Oslo Accident and Emergency Outpatient Clinic, City of Oslo Health Agency, Oslo, Norway; 11 Centre for Patientcentered heart and lung research, Department of cardiothoracic surgery, Oslo University Hospital, Oslo, Norway; 12 Oslo Center for Biostatistics and Epidemiology, Oslo University Hospital, Oslo, Norway; 13 Department of Rehabilitation Medicine, University of Washington, Seattle, Washington, United States of America; 14 Monash Epworth Rehabilitation Research Centre, School of Psychological Sciences, Monash University, Melbourne, Australia; University of South Australia, AUSTRALIA

## Abstract

**Purpose:**

Patients exposed to acute injury or illness are at increased risk of developing mental health disorders, and at the same time, mental health disorders increase the risk for injury and illness. This study aimed to determine the rate and onset of mental health disorders in a mixed patient group receiving inpatient specialized rehabilitation following acute physical injury or illness and to assess whether mental health disorders emerged before or after the injury or illness.

**Methods:**

Patients were recruited over a one-year period (2020–2021) during inpatient rehabilitation. To follow the patients` lifetime history of psychiatric morbidity, mental health disorders, including substance use disorders, were assessed using the M.I.N.I Plus structured diagnostic interview over two periods: 1) retrospective report of mental health disorders before injury or illness and 2) mental health disorders present during rehabilitation. In the latter case, we also took into account whether the condition was present at the time of injury or illness. Demographic and injury data were retrieved from medical charts, patient interviews, and the Oslo University Hospital Trauma Registry.

**Results:**

The study included 130 patients, of whom 49% had a lifetime history, and 38% met the diagnostic criteria for one or more mental health disorders during inpatient rehabilitation. Specifically, the vast majority (72%) of patients with a current disorder had the condition already at the time of injury or illness. Only 5% developed a mental health disorder after injury or illness without having a lifetime history.

**Conclusion:**

Mental health disorders are common and often predate patients physical injury or illness. Assessing patients` mental health in the sub-acute phase, without considering their mental health history, and especially their mental state at the time of injury or illness, may lead to an overestimation of injury or illness’s impact on mental health.

## Introduction

Individuals with complex rehabilitation needs after severe physical injury or medical illness often experience long-lasting physical and psychosocial health challenges [1]. In the rehabilitation setting, those who present with mental health disorders (MHD), including substance use disorders (SUD), constitute a particularly vulnerable population [2–4].

Firstly, persons with MHD are at increased risk of somatic illness and injury [5–7]. A major contributing factor to physical injury is being under the influence of psychoactive substances [8–10], and both acute and chronic consequences of substance use are common causes for admission to intensive care units [9]. Importantly, while some patients may only be under the influence of psychoactive substances at the time of injury or illness, there is a high prevalence of SUDs in the injury population, suggesting long-term patterns of harmful use or dependence [9–11].

Secondly, there is a high prevalence of MHDs among individuals who have experienced physical injury or illness across injury etiologies [6,12–17]. Of interest, one study of persons with moderate to severe traumatic brain injuries found that 62% met the criteria for an MHD during the first year post-injury [18]. Depression rates following spinal cord injury range from 20–40% when assessed with diagnostic criteria [14,19], aligning with the 27−29% rate observed after traumatic brain injuries [20,21] and the 15−50% rate in patients with various medical conditions [17]. Studies employing self-reported assessments for depressive symptoms have indicated even higher prevalence rates [22]. Similarly, after an illness or injury, the prevalence of posttraumatic stress disorders have been found to range from 4%−13% [23,24], while using self-reported measures increases this number to 8–34% [15,25,26]. The large variation in prevalence estimates, likely due to methodological differences across studies [20,27] presents a challenge. Most studies have relied on self-reported symptom measures, which lack the diagnostic specificity of clinician-administered interviews and may misattribute somatic symptoms to mental distress [17]. This issue is particularly important in medical populations. Moreover, studies that have used diagnostic criteria have typically examined specific MHD’s in distinct injury etiologies, potentially excluding a substantial portion of patients, leading to an incomplete understanding of the full spectrum of MHDs in the rehabilitation setting. One exception to this is a study that examined the burden of MHDs in a mixed rehabilitation population, and found a 40% prevalence rate of MHDs, excluding SUDs [23].

Finally, the presence of MHDs during rehabilitation has been associated with adverse functional outcomes post-injury [3]. Notably, MHD seems to be a primary risk factor for suicidal ideation and suicide in patients with various physical health conditions [28,29], including acquired brain injuries [30,31] and spinal cord injuries [32–34].

Given the substantial impact of MHDs on rehabilitation outcomes, evaluating mental health during rehabilitation is essential. However, MHDs remain under-identified and undertreated during both the acute [35] and chronic phases following an injury [36,37] or illness [38].

Of further importance, premorbid MHDs have been identified as key predictors of patients’ mental health post-injury or illness [18,22,27]. Despite this, few studies have incorporated patients’ history of premorbid MHDs when evaluating mental health after an injury or illness during inpatient rehabilitation. Moreover, with some exceptions [6,22], few studies, have taken into account the broad spectrum of patients’ mental health at the time of injury or illness. Ignoring mental health history, particularly the status at the time of injury or illness, may lead to an overestimation of the impact of the injury or illness on mental health [27]. In reality, the causal relationship is more uncertain and may operate in both directions [6].

In summary, a substantial knowledge gap remains regarding the burden and temporal onset of MHDs in patients in need of complex rehabilitation after severe physical injury or illness. To address this, the following research question is addressed in this paper:

1. What is the rate and temporal onset of mental health disorders in patients receiving specialized inpatient rehabilitation after acute illness or traumatic injury?

This study investigated the burden and temporal onset of MHDs in a mixed patient population while they were receiving specialised inpatient rehabilitation after acute illness or traumatic injury. To follow the patients` life-time history of psychiatric morbidity, MHDs were assessed over two periods: 1) retrospective report of MHDs before injury or illness and 2) MHDs present during rehabilitation. In the latter case, we also took into account whether the condition was present at the time of injury or illness, or if it developed later.

We hypothesized that there would be a high burden of lifetime MHDs as well as MHDs present during rehabilitation. Furthermore, we anticipated that there would be a strong association between pre- and post-injury or illness mental health and that many patients with MHD during rehabilitation already had the condition at the time of injury or illness.

## Materials and methods

### Context, recruitment and data collection procedures

This study employed a prospective cohort design, with retrospective collection of baseline data. Participants were assessed during their inpatient rehabilitation stay at Sunnaas Rehabilitation Hospital (SRH). SRH provides specialized rehabilitation to the South-Eastern region in Norway, which serves approximately half of the country`s population, and plays an important role in rehabilitation research. SRH is a civilian hospital and part of the public and universally accessible health care in Norway. Recruitment of participants spanned from January 1, 2020, to February 28, 2021, with a two months break during the beginning of the COVID-19 pandemic. In order to identify eligible patients, members of the research group worked closely with the treating physician at SRH to enroll patients during their inpatient rehabilitation stay. After written informed consent was obtained, an experienced clinical psychologist conducted the clinical interviews and obtained the questionnaire data. A physician (author CU) searched the medical records for the relevant information. Additionally, a nurse and a social worker collected sociodemographic information and details regarding patients’ functional independence from their charts.

#### Inclusion and exclusion criteria.

Inclusion criteria required age ≥ 18 years and admission to inpatient sub-acute specialized rehabilitation at SRH following a acute injury or illness; receiving treatment in the Departments of Traumatic Brain Injury, Spinal Cord Injury, or Multitrauma, Neurology, and Burns. All participants had to meet the International Classification of Diseases, 10th. Revision (ICD-10) criteria for complex rehabilitation code Z50.80 [39]. Exclusion criteria were admission due to a preexisting chronic or progressive condition. Patients with moderate to severe brain injuries, assessed with a Glasgow Coma Scale score 3–12 [40], were considered for inclusion when emerged from Post Traumatic Amnesia and able to provide informed consent. This means they had regained orientation to time, place, and personal circumstances, as assessed with the orientation questions in the Galveston Orientation and Amnesia Test [41].

### Ethics

Participants with MHDs in addition to physical injury are vulnerable in the rehabilitation context, and ethical issues such as timing and duration of assessment according to psychological and somatic status was considered. Diagnostic results from the psychiatric assessment were not recorded in patient charts unless considered important for clinical management, and only with explicit participant consent. This procedure was described in consent forms and presented to the Ethical committee. All participants provided written informed consent. For patients with acquired brain injury but intact consent capacity, the form included a separate item granting permission to contact next of kin. No secondary use of data occurred. Clinical care was unaffected, as psychologists on the clinical team conducted regular assessments and provided treatment. The study was evaluated to pose no risk to individuals or privacy. The study was approved by the Regional Committee for Medical and Health Ethics in South-East Norway (reference number 2019/1284), and data protection issues were considered by the data protection officer at SRH and the Norwegian Center for research Data (NSD), (reference number 875220).

### Measurements

#### Demographics and medical variables.

We obtained demographic information such as sex, age, marital status, and level of education through patient interviews. Injury-related information, such as ICD-10 codes, cause and date of illness or injury, and number of days before admission to inpatient rehabilitation was obtained from the medical file. For patients with a traumatic injury, who were initially admitted to Oslo University Hospital, Ullevål – the regional trauma center of Southeastern Norway -, the New Injury Severity Score (NISS) [42] and GCS score [40] were both extracted from the Oslo University Hospital Trauma Registry. These data were obtained on December 17, 2021. The NISS in this study is derived from the Abbreviated Injury Scale 2008 edition (AIS-08). Each anatomical injury is assigned a unique 6-digit numerical code in addition to the AIS severity score where the former ascribes body region and anatomical structure and the latter digit ascribes anatomical severity on a scale from 1 to 6 [43]. The NISS incorporates the three most severe injuries by summing the square of the three highest AIS severity scores independent of injured body regions, ranging from 1 to 75 (maximal injury) [42], NISS scores greater than or equal to 9 is considered as moderate-to-severe injury. Glasgow Coma Scale (GCS) scores [40] were recorded for patients with an acquired brain injury to measure level of consciousness on admission to the trauma centre. The GCS score ranges from three (totally unresponsive) to 15 (fully responsive) [40]. The NISS and GCS scores were both extracted from the Oslo University Hospital Trauma Registry. We categorized patients` primary medical diagnoses into five categories: 1. spinal cord injury, 2. acquired brain injury, 3. multitrauma (the presence of two or more injuries affecting different physical regions or organ systems), with and without amputations, 4. polyneuropathy, and 5. “other medical diagnoses”. Other medical diagnoses included post-infectious conditions following COVID-19, amputations following a disease, injury without multitrauma, radiculopathy, burn injuries, an unspecified paralytic syndrome (ICD-10 code G83.9), muscle ischemia, and fractures without multitrauma.

#### Clinical assessment of mental health disorders.

**Fig 1:**
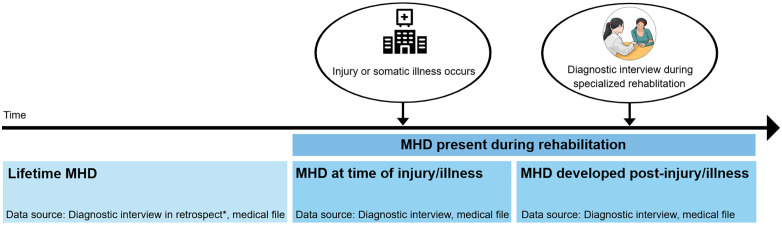
Assessment of mental health disorders at two time periods.

Gold-standard diagnostic interviews and a transdisciplinary expert consensus approach was used to establish the burden and temporal onset of MHDs in this mixed patient population. To follow the patients` life-time history of psychiatric morbidity, MHD was assessed over two periods: 1) retrospective report of MHD before injury or illness and 2) MHD present during rehabilitation. For the latter, it was also considered whether the condition was present at the time of injury/illness (less than one month before injury/illness) or developed after (se Fig 1).

Patients lifetime history of MHD was assessed retrospectively based on information from the diagnostic interview, in addition to pre-injury psychiatric diagnosis or other mental health information recorded in patients’ medical files. Information from acute hospital and rehabilitation medical charts was utilized alongside diagnostic interviews, to determine ongoing MHDs and whether an MHD developed before or after injury or illness. If an Axis I disorder was mentioned in medical records, but the patient did not meet the criteria for a current MHD based on a structured clinical interview, we classified it as a lifetime MHD. This also applied to patients taking antidepressant medication without ongoing symptoms. All patients’ lifetime diagnoses were specified as either present or in remission at the time of injury/illness. If no MHDs were present during this period, they were classified as being in full remission. If they had one or more MHDs in remission but at least one MHD present at time of injury or illness, they were classified as being in partial remission; and those with no disorders in remission were classified as not in remission at time of injury or illness. On the other hand, when reporting the occurrence of distinct lifetime and current diagnoses, only MHDs that were in remission at least one month prior to the injury or illness were classified as lifetime diagnoses. Patients meeting diagnostic criteria one month prior to injury or illness or at any time during the rehabilitation stay were considered to have a current MHD.

For an SUD to be in remission, there needed to be no symptoms of harmful use or addiction present in the previous 12 months. For conditions with expected stability across the lifespan, such as personality disorders, Asperger’s, and attention-deficit hyperactivity disorder, diagnoses were recorded. There were no instances where these diagnoses were noted in patient charts and the research team disagreed. While all patients provided informed consent to take part in the study, those with acquired brain injuries may have had cognitive impairments which could influence their ability to provide accurate information. As a result, these patients were asked to consent to their next of kin being interviewed for diagnostic information to validate the accuracy of patient-reported information both pre- and post-injury. All interviews were conducted by psychologists with extensive diagnostic training. The research team made the final diagnostic decision based on consensus discussions in a multidisciplinary research group consisting of experienced neuropsychologists, clinical psychologists, trauma nurse, medical specialists in psychiatry, neurology and physical medicine and rehabilitation. This ensured that adequate differential diagnostic considerations were made. Careful consideration was made to ensure that diagnoses were set according to ICD-10 criteria, and that normal psychological reactions to severe life-changing circumstances were not unduly diagnosed as MHDs, i.e., a conservative approach was applied.

#### Assessment tools.

Diagnostic interviews were performed with the gold-standard semi-structured interview M.I.N.I. Plus Neuropsychiatric Interview, version 5.0.0, which is based on ICD-10 criteria and has good reliability for all included diagnoses [44]. The M.I.N.I Plus assesses current and past MHDs, and we modified it to determine whether the MHDs fit into the study-specific timeframes. In addition, due to difficult differential diagnostics in the post-acute phase of somatic injury/illness, pre-but not post-injury or illness somatoform disorders (F45) were diagnosed. For the same reason, we excluded assessments of current personality disorders (F06.2), but any pre-morbid personality disorders documented in the medical file were included. Furthermore, we included diagnostics of pre-injury/illness non-organic insomnia (F51.0) and use of anabolic-androgenic steroids (F55.5). Current organic mental health disorders (F00-09) were recorded but were not included in the analysis. All patients were asked about previous and ongoing suicidal ideation and previous suicide attempts as formulated in the M.I.N.I Plus.

In addition to the M.I.N.I. Plus interviews, we conducted risk assessments for substance use to obtain standardized information about patients’ use of alcohol and drugs. We utilized the Alcohol Use Disorders Identification Test (AUDIT) and the Drug Use Disorders Identification Test (DUDIT). The AUDIT was specifically used to assess harmful patterns of alcohol use in the 12 months prior to injury/illness, as well as earlier in life. The AUDIT is a 10-item questionnaire, and responses to each item are scored from 0 to 4, giving a maximum score of 40. Score of ≥8 points for male [45], and ≥6 for females [46] were used as the cut-off point for high risk alcohol consumption. Cronbach’s alpha coefficient has previously been found to be 0.86 [47]. The DUDIT was used to assess harmful patterns of substance use in the 12 months prior to injury, as well as earlier in life. DUDIT is an 11-item questionnaire, and responses to each item were scored from 0 to 4, giving a maximum score of 44. A score greater than or equal to 2 points is the cut-off point for high risk drug use in females; for men the cut-off point is greater than or equal to 6 [48]. Cronbach`s alpha coefficient of 0.94 has previously been found [49].

#### Functional independence measure.

The Functional Independence Measure (FIM) [50] was used to assess patients` functional levels and need for assistance during the first three days after admission to rehabilitation. The FIM assesses patients across 18 areas (13 motor and 5 cognitive domains), including self-care, bowel and bladder management, mobility, communication, cognition and psychosocial adjustment. Each of the 18 items is rated from 1 (total assistance) to 7 (complete independence) depending on the patient’s need for assistance, resulting in a total score between 18 (total dependence) and 126 (total independence). FIM has been found to be valid and reliable for measuring functional outcomes in various patients groups [51]. FIM has also been found to have excellent reliability with test–retest, interrater reliability, and internal consistency above 0.90 [52]. The FIM total score was further categorized into three levels of dependence severity: mild (109–126); moderate (72–108), and severe (< 72) [53]. We used FIM version 5.0, the most recent Norwegian version. FIM scores were derived from clinical charts and were retrospectively verified by consensus between two authors (authors ASS, ML, NA or SLH).

### Statistical analysis

IBM SPSS, Version 29, was employed to conduct data analyses. Demographic and medical data, as well as the proportion of MHD, were summarized as medians with interquartile range (IQR), or proportions if not stated otherwise. To examine associations between relevant variables potentially associated with medical diagnoses, the following five variables were examined: 1. sex, 2. age, 3. Functional Independence Measure (FIM), 4. length of stay, and 5. trauma/non-trauma etiology. All were chosen due to their potential clinical and theoretical relevance in relation to mental health disorders. We employed the Kruskal-Wallis test to detect disparities in distribution in age, FIM scores, and length of stay between the five medical diagnoses. To determine whether there was an association between the five medical diagnoses and lifetime MHD and MHD present during rehabilitation we implemented either the chi-squared test (χ²) or the Fisher-Freeman-Halton exact test as appropriate. We investigated whether there were any differences in MHD between patients with traumatic compared to nontraumatic etiology and sex using the same approach as described above. Pairwise comparisons were further conducted to identify group differences using the Bonferroni correction for multiple tests. The corrected Bonferroni p-value threshold was set at P < .005 (0.05/10) for the categorical post-hoc pairwise tests. Effect sizes were calculated by Cramér’s V, r, η², OR (odds ratio) CI (confidence intervals), and Bayes factors, as appropriate. For Cramér’s V, and r, small effects were defined as 0.1, medium effects were 0.3, and large effects were 0.5. For η², small effects were defined as 0.01, moderate effects as 0.06, and large effects as 0.14. Bayes factors were interpreted based on established thresholds, where Bayes factors between 1 and 3 indicate small support, values between 3 and 10 indicate moderate support, and values greater that 10 indicate strong support for the alternative hypothesis (Jeffreys, 1961, as cited in [54], p. 129). A p-value of less than 0.05 was considered significant. Missing data are reported, and only complete case analyses are presented.

## Results

### Participants

**Fig 2:**
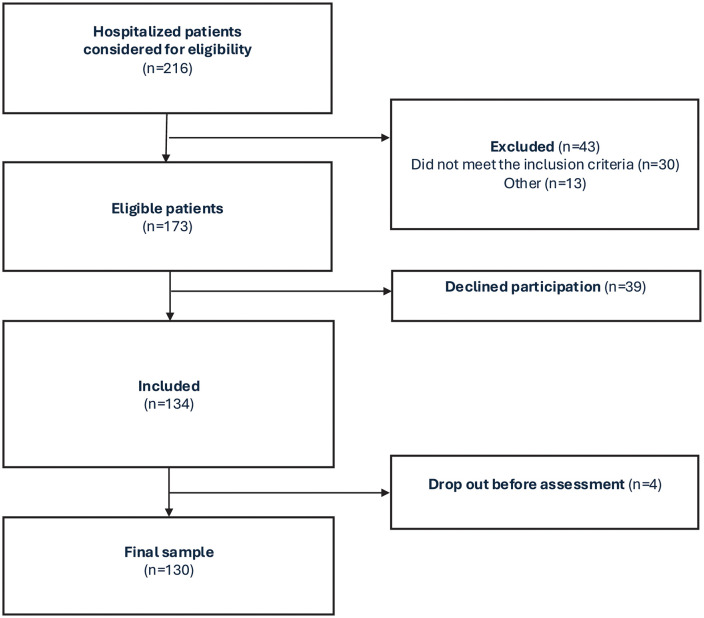
Patient flowchart.

Of 173 eligible patients, 134 were included, of whom 130 (77%) completed the assessment (Fig 2). Of the 33 patients with acquired brain injury, 24 consented to a close family member being interviewed.

The majority of participants were male (90/130 = 69%), and the median (IQR) age was 52 (39–63), (see Table 1). Total FIM scores at admission ranged from 36 to 125, with a median (IQR) score of 96 (72–111), with 2/3 of the sample exhibiting moderate to severe dependency. The majority, 63% (82/130), had experienced a traumatic injury. All traumatic injuries had NISS scores greater than or equal to 9, indicating moderate-to-severe injury.

**Table 1:** Sociodemographic and medical description of the total sample (n = 130).

Sociodemographic variables	n (%)	median (IQR)	Range
Age		52 (39-63)	18-77
Sex, male	90 (69%)		
*Relationship status the time of illness or injury*			
Married/domestic partner	76 (58%)		
Widowed/divorced/single	54 (42%)		
*Education*			
≥ 13 years	71 (54%)		
< 13 years	59 (46%)		
*Source of income at time of injury*			
Work/education	64 (49%)		
Retirement pension	21 (16%)		
State financed social benefits	42 (33%)		
Other sources of income^a^	3 (2%)		
**Injury/illness related variables and function during rehabilitation**	**n (%)**	**Median (IQR)**	**Range**
Days since injury before admission to SRH^b^		29 (15-51)	7-157
Length of stay at SRH (days)		66 (45-93)	13-241
*Injury/illness etiology*			
Spinal cord injury	48 (36%)		
Acquired brain injury	33 (25%)		
Multitrauma	23 (18)		
Polyneuropathy	11 (9%)		
Other medical diagnosis^c^	15 (12%)		
*Functional independent measure* ^*d*^			
FIM at admission SRH, total score		96 (72-111)	36-125
Mild	40 (31%)		
Moderate	58 (44%)		
Severe	32 (25%)		
*Traumatic injury severity*			
NISS^e^ for traumatic injuries	64 (78%)	26 (22-41)	9-66
GCS^f^ for traumatic brain injury	25 (89%)	7 (4- 13)	3-15

^a^Other sources of income include living at home with parents, living off savings, or being supported by a spouse.

^b^SRH = Sunnaas Rehabilitation Hospital.

^c^Other medical diagnoses include post-infectious conditions following COVID-19, amputations following a disease or injury without multitrauma, radiculopathy, burn injuries, an unspecified paralytic syndrome, muscle ischemia, and fractures without multitrauma.

^d^FIM = Functional Independence Measure.

^e^NISS = New Injury Severity Score. NISS scores registered for 64 of 82 trauma patients. NISS scores for 18 patients are missing, due to admission to other trauma hospitals than Oslo University Hospital, Ullevål (in Norway or abroad). NISS ≥ 9 is considered as moderate-to-severe injury.

^f^GCS = Glasgow Coma Scale. GCS scores registered for 25 of 28 patients with traumatic brain injury on admission to Oslo University Hospital, Ullevål. GCS scores for three TBI patients are missing due to injuries abroad.

There was a significant sex distribution between the diagnostic groups (X^2^) = 10.09, p = 0.035). Cramér’s V-value of 0.29 supports a moderate, and Bayes factor 2.45 supports weak support for this association. However, post-hoc pairwise comparisons showed no significant differences between the diagnostic groups after Bonferroni correction. Although both the brain injury and spinal cord injury groups had a higher proportion of males compared to the other group, this difference approached significance (p = 0.005 for both), (see Table 2 for details). There was a significant difference in distribution of age-scores between the five diagnostic groups (H) = 17.06 (4), p = 0.002). Post hoc pairwise comparison showed a significant higher age for those with spinal cord injuries compared to those with multitrauma (median (IQR): 61 (49–66) versus 45 (31–53), (H) = 37.24, adj. sig. p = 0.001). The effect size (r = 0.46) indicates a moderate to strong effect (see Table 2). There were also a difference in the distribution of FIM-scores between the five diagnostic groups (H) = 10.51(4), p = 0.033). Pos hoc pairwise comparison showd that patients with spinal cord injuries had lower FIM-scores, indicating higher dependency levels, compared to patients with acquired brain injuries (median (IQR): 80 (57–106) versus 105 (77–119), (H) = 24.42, adj. sig. p = 0.041). The effect size (r) indicates a moderate effect (r = 0.32).There was an uneven distribution of traumatic and non-traumatic etiologies between medical diagnostic groups: While 85% (28/33) of the brain injuries were traumatic, spinal cord injuries had approximately equal proportions of traumatic 54% (26/46) versus non-traumatic 46% (22/46) etiologies. Naturally, all multitrauma and were traumatic and all polyneuropathies and a majority of other medical diagnoses were non-traumatic 67% (10/15). There was no significant difference in length of stay between the five diagnostic categories, (H) = 5.28 (4), p = 0.260), and the effect size was small (η²)= 0.01).

**Table 2:** Sex, age, functional independence scores at admission and length of rehabilitation stay for the five medical groups.

Primary medical diagnoses	Sex male/n, (%)	Age median (IQR)	FIM^a^ at admission SRH^b^, total score median (IQR)	Length of stay at SRH^b^ (days) median (IQR)
Spinal cord injury (SCI)	35/48 (73%)	61 (49–66) ^***(MT)^	80 (57–106) ^*(ABI)^	76 (44-105)
Aquired brain injury (ABI)	25/33 (76%)	51 (38-62)	105 (77–119) ^*(SCI)^	61 (50-69)
Multitrauma (MT)	18/23 (78%)	45 (31–53) ^***(SCI)^	106 (88-111)	70 (39-104)
Polyneuropathy	7/11 (64%)	51 (42-60)	107 (56-115)	64 (30-120)
Other diagnoses	5/15 (33%)	47 (28-63)	102 (87-108)	63 (29-90)

*p = 0.05, **p = 0.025, *** = p < 0.001.

^a^FIM = functional independence measure.

^b^SRH = Sunnaas rehabilitation hospital.

### Rates and temporal onset of mental health disorders

**Fig 3:**
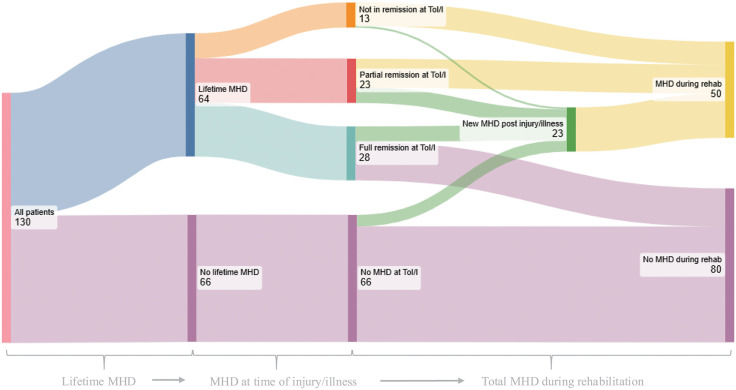
Sankey diagram of patients’ mental health status over time. The Sankey diagram (created using SankeyMatic.com) illustrates the number of patients with a lifetime history of MHD or MHD present during rehabilitation and specifies whether the patient had an MHD present at the time of injury or illness. Thicker lines represent higher proportions of patients transitioning between mental health states. Read from left to right.

Fig 3 illustrates the proportion of all patients with MHD during the two time periods. A total of 38% of patients (50/130) met criteria for at least one MHD during their rehabilitation stay. Of all patients with a current disorder, 56% (28/50) qualified for one diagnosis only, and 44% (22/50) qualified for two diagnoses or more. Notably, 72% (36/50) of those with a current MHD already had an MHD at the time of injury or illness, while 28% (14/50) developed their MHD after the injury or illness. Importantly, only 5% (6/130) of the sample developed an MHD during rehabilitation without having a life-time history of mental illness.

In addition to those with a current MHD, approximately half of the patients, 49% (64/130), had a lifetime history of one or more MHD. Of these, 44% (28/64) were in full remission at the time of injury or illness; 36% (23/64) were in partial remission, meaning they had one or more MHDs in remission but at least one MHD still present at the time of injury or illness. The remaining 20% (13/64) had all of their lifetime MHDs still present at the time of injury or illness.

### Distribution of mental health diagnosis

In addition to looking at rates of MHD in individual persons, we also explored the occurrence of distinct diagnoses, see Table 3 for details. During rehabilitation, anxiety disorders were most common with a 37% (30/81) occurance, followed by mood disorders with a 27% (22/81) occurance and substance use disorders amounting to a 20% (16/81) occurance. Percentages are calculated based on the total number of mental health diagnoses presented during rehabilitation (n = 81), not the full sample (n = 130). The most common MHDs developing after injury or illness, were anxiety disorders and mood disorders, each accounting for 50% of new diagnoses (12 out of 24). Of specific disorders, recurrent depression, followed by adjustment disorder, major depressive episode, and post-traumatic stress disorder (PTSD) were the most common disordes to develop post-injury or illness (see Table 3). One patient received two diagnoses. For the six patients without a lifetime history of MHD, four developed a depressive episode and two developed an adjustment disorder. Additionally, two patients with acquired brain injury developed an organic affective disorder (F06.3), and organic paranoid disorder (F06.2). These were excluded from the table and subsequent analyses as the conditions were directly caused by the injury and subsided during rehabilitation.

**Table 3:** Distribution of mental health disorders according to the ICD-10. The percentages in each diagnostic category represent the numbers of MHDs in relation to the total number of MHDs for that time period.

MHDs according to ICD-10	Lifetime MHDs^a^	MHDs during rehabilitation		
		*Present <1 month pre-injury/illness*	*Developed post-injury/illness*	*Total during rehabilitation*
**Mood disorders (F30-39)**	**33 (46%)**	**10 (18%)**	**12 (50%)**	**22 (27%)**
Major depressive disorder	16	0	5	5
Recurrent depressions	16	9	7	16
Bipolar disorder	0	1	0	1
Other mood disorders^b^	1	0	0	0
**Anxiety disorders (F40-49)**	**22 (31%)**	**18 (32%)**	**12 (50%)**	**30 (37%)**
Post-traumatic stress disorders (PTSD)	6	3	4	7
Adjustment disorders	3	3	6	9
Social phobia	5	3	0	3
Generalized anxiety disorder	0	4	0	4
Panic disorder	2	1	0	1
Dissosiative and somatoform disorders^c^	2	1	2	3
Other anxiety disorders^d^	4	3	0	3
**Substance use disorder (F10-F19)**	**7 (10%)**	**16 (28%)**	**0**	**16 (20%)**
Alcohol: harmful use/dependence	4	10	0	10
Drug^e^: harmful use/dependence	3	6	0	6
**Psychotic disorders**^f^ **(F20-29)**	**0**	**1 (2%)**	**0**	**1 (1%)**
**Eating disorders, sleep disorders and abuse of non-dependent substances**^**g**^ **(F50-59)**	**9 (13%)**	**3 (5%)**	**0**	**3 (4%)**
**Personality disorders**^**h**^ **(F60-69)**	**0**	**4 (7%)**	**0**	**4 (5%)**
**Development disorders**^**i**^ **(F80-89)**	**0**	**1 (2%)**	**0**	**1 (1%)**
**Attention deficit disorders (F90-98)**	**0**	**4 (7%)**	**0**	**4 (5%)**
**All disorders summarized**	**71 (100%)**	**57 (100%)**	**24 (100%)**	**81 (100%)**

^a^Lifetime MHD, in remission >1 month pre-injury/illness.

^b^Other mood disorders refer to (F32.9).

^c^Dissociative and somatoform disorders include (F44.7, F44.8 and F45.4).

^d^Other anxiety disorders include (F40.2; F41.2; 41.8 and 41.9).

^e^Substance use disorders include (F11.2, F13.2, F14.2 and F19.2).

^f^Psychotic disorders refer to (F22.9).

^g^Eating disorders include (F50.1, F50.4, F50.8 and F50.9, F51.0, F55.5),

^h^Personality disorders include (F60.2, F60.3, F60.6 and F60.7),

^i^Development disorders refer to (F84.5).

At the time of injury or illness, anxiety disorders were most common, affecting 32% (18/57), of patients, followed by substance use disorders, which affected 28% (16/57) of patients; with dependence and harmful use of alcohol was the most frequent diagnosis, followed by dependence on multiple psychoactive substances (F19.2) and opioid dependence (F11.2). Mood disorders affected 18% (10/57) of patients, with recurrent depression being the most common.

For lifetime MHDs in remission at the time of injury/illness, mood disorders were most frequent, affecting 47% (33/71), followed by anxiety disorders at 31% (22/71). PTSD was the most common anxiety diagnosis, and 10% (7/71) of patients had an SUD in sustained remission, of which five also had a history of substance-induced psychosis. In addition, four participants reported previous use of anabolic-androgenic steroids, and one participant was using anabolic-androgenic steroids at time of injury or illness.

#### Suicidal ideation and previous suicidal attempts.

A total of 18% (23/130) of patients reported suicidal ideation during rehabilitation. Of these, 87% (20/23) had an MHD. Furthermore, 10% (13/130) of the sample had experienced a previous suicide attempt, while three patients reporting multiple attempts. All had a lifetime history of MHDs. Some, 38% (5/13), of those with a previous suicide attempt reported suicidal ideation during rehabilitation.

### Mental health disorders across medical groups

There were a significant difference in the distribution of MHDs present during rehabilitation between the five diagnostic medical groups (X^2^) = 15.73 (4), p = 0.003). A moderate effect size (Cramér’s V = 0.35) and a moderate Bayes factor (BF = 5) both provide support for this association. MHDs were observed in 64% (7 out of 11) of patients with polyneuropathy during rehabilitation, followed by 60% (9 out of 15) among those with other diagnoses, and 57% (12 out of 23) in patients with multitrauma. In contrast, the rate of MHDs was lower among patients with acquired brain injury (33%, 11 out of 33) and those with spinal cord injury (21%, 10 out of 48). Post-hoc pairwise comparisons showed significant differences between patients with spinal cord injury and those with multitrauma (p = 0.003), as well as between patients with spinal cord injury and other diagnoses (p = 0.004). The difference between patients with spinal cord injury and those with polyneuropathy was just on the threshold (p = 0.005). All p-values for the pairwise comparisons are Bonferroni corrected. Group differences are presented in Table 4, which provides the odds ratios (OR) and confidence intervals (CI) for MHDs for each medical group, within the spinal cord injury group serving as the reference category. However, in the acquired brain injury group, 21% (7/33) had SUDs, with all cases being related to alcohol use. In the multitrauma group, 17% (4/23) had SUDs, with all of these related to drug use (F11.2, F13.2, F14.2 and F19.2). The limited sample sizes within each group hinder a more comprehensive analysis of the various MHDs in the different medical groups.

**Table 4:** Distribution of MHD during rehab for the five medical groups.

Primary medical diagnosis	Number of patients with MHD during rehabilitation (n, %)	OR	95% condidence interval (CI)
Spinal cord injury (SCI)	10/48 (21%)	1.00 ^(reference)^	_
Aquired brain injury (ABI)	11/33 (33%)	1.90	0.70–5.19
Multitrauma (MT)	12/23 (52%)	4.15	1.14–12.14
Polyneuropathy	7/11 (64%)	6.65	1.62–27.30
Other diagnoses	9/15 (60%)	5.70	1.64–19.81

There was no significant difference in distribution of lifetime MHDs between the five medical groups (X^2^) = 6,07(4), p = 0.198. Both Cramér’s V (0.22), and Bayes factor (0.42) support a low association. There were no significant differences in distribution of MHD rates during rehabilitation, independent of whether the participants had an acute illness or a traumatic injury (X^2^) = 0.90 (1), p = 0.343. Both Cramér’s V value (0.08), and Bayes factor (0.47) support a low association. However, a significant difference was found between sex and MHDs during rehabilitation (X^2^) = 6.68 (1), p = 0.010, with women having over 2.5 times higher odds of having an MHD during rehabilitation compared to men (OR = 2.71, CI 95% 1.26–5.82).

## Discussion

The aim of this study was to investigate the burden and temporal onset of MHDs in a mixed patient population after acute illness or traumatic injury. This study shows that 49% of patients admitted to specialized rehabilitation following acute physical injury or illness had a history of MHD, verified either through the retrospective part of the diagnostic interview or from medical charts. Additionally, 38% met the diagnostic criteria for one or more MHD during their inpatient rehabilitation stay. Importantly, 72% of patients diagnosed with an MHD during rehabilitation already had the disorder at the time of injury or illness, while only a 5% developed their first MHD afterward. This finding is important because prior studies among patients in rehabilitation rarely assessed MHDs at or before the time of injury, potentially overestimating the impact of injury or illness on mental health.

### High rates of MHD across medical groups

The number of patients who met the criteria for an MHD during their rehabilitation stay clearly exceeded estimates for the general Norwegian population, where the one-year prevalence is between 16 and 22% according to proxy surveys [55]. However, the present study’s rate matches the 40% rate from the one previous study that used diagnostic interviews to assess MHD during rehabilitation in a mixed patient population [23]. On the other hand, that study did not include SUDs. When excluding SUDs in our analysis the rate reduced from 38 to 32%.

Our study aligns with earlier findings that many patients with MHDs already have had their conditions before their injury or illness. For instance, approximately 39% of patients with orthopedic polytraumas had a pre-injury MHD [6], whereas 16% of patients with traumatic brain injuries had a pre-existing depression [22]. However, neither of these studies assessed patients based on symptom-spesific criteria; instead they relied on other indicators, such as registered diagnoses or whether they had received counseling or antidepressants within the six months preceding the injury. Therefore, conditions that we potentially would classify as lifetime conditions might have been included as current disorders in these studies. Still, the current study highlights the closeness between premorbid and current mental health in the injury population.

### Common diagnoses and group variations

Mood, anxiety, and substance use disorders were the most frequently observed diagnostic categories, both over the lifetime and during rehabilitation. This is consistent with findings from studies in both the general population [56] and somatic patient populations [18,23,24]. Anxiety disorders were most prevalent during rehabilitation, while mood disorders dominated in a lifetime perspective. Depression, adjustment disorders and PTSD were the most frequently occurring disorders post injury or illness. The 3% rate of PTSDs developed after injury or illness in the current study was lower than the 4–13% rates reported in similar studies using diagnostic interviews [23,24]. However, if we were to include patients with PTSD at the time of injury, the rate would increase to 5%. This suggest that previous studies may have overestimated the actual rate of PTSD emerging post injury or illness, as few studies have distinguished between mental health conditions emerging before or after injury or illness. Follow-up studies are important to establish to what degree adjustment disorders progress to more severe conditions like PTSDs over time.This was indeed found in Gould et al which suggested that this progression may be linked to patients’ enhanced ability to identify and articulate psychiatric symptoms over time [24]. Depression affected 16% of patients, which is lower than the 20–43% rates reported in studies of persons with spinal cord injuries [14], traumatic brain injuries [18,24], and mixed populations [23]. However, in the current study, patients were classified as having a depression only if they adhered to diagnostic criteria at the time of assessment. Depressive symptoms in remission were not classified as current depression, even if patients were still receiving treatment. Our findings indicating a 12% rate of patients with SUDs at the time of injury or illness also seem reasonable given that SUDs are a common risk factor for injury or illness. Given that patients with SUDs are found to have more MHDs and use twice the amount of opioids for chronic pain compared to those without SUDs [57], there is a need to explore opioid use among patients in specialized rehabilitation. Moreover, this is the first study to explore the use of anabolic androgenic steroids in a medical inpatient cohort. Among male patients, 6% reported lifetime use, twice the rate of the general Norwegian population [58]. Mapping anabolic androgenic steroids use is important, as body image issues arising during rehabilitation may contribute to continued use [59], posing risks of mental and physical side effects [60], which may have negative impacts on rehabilitation. In summary, although the 38% rate of MHDs among the patients group may appear high, this rate is lower than comparable studies when split into specific diagnoses. Our study revealed a low number of persons with ongoing psychosis (1%), consistent with previous studies [18]. However, referral bias cannot be ruled out, as patients with a known psychosis may have reduced access to specialized rehabilitation services. Recruiting patients directly from the acute hospital could have mitigated this limitation. Nonetheless, the primary objective of the current study was to study a typical rehabilitation cohort.

The current study found that the rate of MHDs during rehabilitationvaried across different diagnostic categories, with notably higher rates among patients with multitraume and other diagnoses compared to those with spinal cord injuries. Despite the limited sample size, these results underscore the importance of including a diverse patient group, as less-studied medical groups may have the highest MHD rates. Although individuals with traumatic brain injuries have previously been hypothesized to experience a higher burden of MHDs, due to the specific impact of brain injury, which is believed to cause more emotional distress than other trauma [18,24], the current study did not find support for this hypothesis in the sub-acute phase. However, a higher rate of alcohol use disorders was found for patients with traumatic brain injuries, consistent with other traumatic brain injury studies [18,61], suggestsong that alcohol-related interventions could hold particular significance after traumatic brain injury. Importantly, no differences were found between patients suffering from an illness or a traumatic injury, supporting previous findings by Carlson et al. [26]. This highlighets the need for further research into the illness population, which is less studied than the trauma population in rehabilitation, in order to better understand their specific needs and ensure necessary follow-up.

### Suicidal ideation

In line with previous studies, we found a strong association between suicidal ideation and MHD, with 87% of patients experiencing suicidal ideation also meeting the criteria for an MHD. Notably, 18% of patients reported suicidal ideation during rehabilitation, which is more than four times higher than the 4% prevalence observed in the general Norwegian population [62]. This aligns with previous studies, which reported suicidal ideation rates in 13% of individuals with spinal cord injuries [33] and 25% of those with traumatic brain injuries [63]. However, these rates are somewhat higher than the 7% reported in a mixed patient group [29]. It is important to note that these studies differ regarding time of assessment. In sum, given the high number of patients experiencing suicidal ideation during rehabilitation, and the recognition of enhanced risk of suicide in this population, necessitates a proactive approach to suicide prevention, especially during the critical period around discharge. Brief suicide prevention interventions delivered in a single in-person encounter in acute care has shown to be effective in reducing subsequent suicide attempts, although the MHD persists [64]. Furthermore, local follow-up is crucial; in cases where it is not accecable at the time of discharge, we recommend that rehabilitation facilities provide temporary support to bridge the transition from institutional care to home dwelling. Telehealth could be a valuable tool by providing flexible and accessible care without significantly straining resources [65]. It is also recommended to develop a safety plan with the patient prior to discharge, with specific emphasis on the development of strategies to address intrusive suicidal ideation. This kind of safety plan has already been identified as important in mitigating the risk of suicide [66].

### Resilience in the rehabilitation population

The fact that the majority, i.e., more than 60% of included patients, did not experience any MHD after injury of illness, and only 5% of the patients (6/130) without a pre-injury history of MHD developed an MHD after the injury or illness, is indicative of a high level of resilience in the rehabilitation population. This aligns with earlier research in spinal cord injury and multi-trauma populations [67], where a resilient trajectory was the most common response (54%) to an severe acquired injury during inpatient rehabilitation. These findings thus underscore previous studies [68] which highlight patiets` remarkable capacity to cope with adverse life events, particularly those with robust premorbid mental health. In addition, a review [69]. found resilience to be the predominant response to potentially tramatic events, with a pooled prevalence rate of 66% across diverse studies and populations. However, there remains a critcal need for follow-up studies extending beyond the sub-acute phase. Future studies should also consider relevant pre- and post-injury stressors that influence adjustment after injury [69] and aim to identify particularly vulnerable subgroups, thereby improving our understanding of patients’ long-term follow-up needs in the aftermath of trauma or illness.

### Strength and limitations

The diagnostic process was a key strength of the study as it relied on both the use of gold standard diagnostic instruments and multidisciplinary consensus to ensure thorough and accurate diagnoses. Acknowledging that the field of psychiatry has faced criticism for being overly dominated by biologically oriented thinking in recent decades [70], we were particularly mindful in this context to emphasize patients’ subjectivity, personal meaning and cultural factors when conducting clinical interviews and assessing symptoms in accordance with diagnostic criteria. This perspective was crucial for addressing the often complex situations and health issues of the included patients. However, while patients’ subjective experiences were valued, structured diagnostic interviews and multidisciplinary consensus ensured that diagnoses met objective ICD-10 criteria, minimizing potential bias. For many, their injury or illness was a traumatic experience, where normal reactions to extraordinary events can be expected. Potentially life-changing experiences can elicit strong emotional reactions like grief, loss, insecurities about the future and pain, particularly important in the rehabilitation setting. We therefore took a conservative approach when diagnosing MHDs, ensuring that diagnostic criteria were met. Our results may thus even underestimate the prevalence of some conditions. Importantly, patients exhibiting symptoms below the diagnostic threshold may still require additional support and care.

Research on a mixed rehabilitation population presents certain limitations. Different etiologies make comparisons of injury severity challenging, which is why we used the FIM to establish a generic measure of functional impairment. The relatively small sample size limited the ability to conduct sub-group analyses on the etiology and development of various mental disorders and we didn’t have enough statistical power to perform adjusted analyses controlling for covariates. The short follow-up period precluded assessment of long-term mental health outcomes. Planned publications of 2–3-year outcome data in the same cohort will address the latter limitation.

The study included patients admitted to specialized in-patient rehabilitation in the sub-acute phase after injury or illness. Hence, the results might not be applicable to patients who were not transferred to specialized inpatient rehabilitation or those with less severe injuries/illnesses. Additionally, we waited to include patients with brain injury until they were able to provide informed consent. Consequently, some individuals with the most severe injuries were not enrolled, which may have introduced recruitment bias. Moreover, this study was conducted during the COVID-19 pandemic, a factor which may have influenced mental stress [71]. However, meta-analyses indicate that the COVID-19 pandemic’s impact on mental health was largely confined to its early months [72,73] and studies on mental stress after the initial period have been inconsistent globally, with overall low effects [74]. In the current study, data collection was paused from March to May 2020, potentially reducing some early pandemic-related effects. However, some patients with somatic conditions may have experienced higher mental stress during the pandemic as they faced greater physical threats. In a study, trauma patients have shown increased stress levels during hospitalization during the pandemic [75], although the increase may reflect acute emotional reactions rather than diagnosable MHDs [76]. Further, the study was conducted in Norway, a country known for strong public trust in healthcare systems, which may also have mitigated the effects of COVID-19 pandemic related stress [76,77]. One study found stable levels of mental health during the early COVID-19 pandemic in Norway [62], reinforcing this argument. In summary, despite the limitations associated with conducting research during a pandemic, these factors suggest that the findings of this study may still be generalizable beyond the pandemic context.

## Conclusions and clinical implications

To our knowledge, this is the first study to use a gold-standard diagnostic interview to assess MHDs in a mixed rehabilitation population with a lifetime perspective, capturing mental health status both during inpatient rehabilitation and at the time of injury or illness. Although a notable minority developed new MHDs, the findings indicate relative stability in mental health, as almost all patients diagnosed with an MHD during rehabilitation either had a lifetime history of MHDs or had their condition at the time of injury or illness. The high rate of patients with MHD across medical groups underscores the need for integrated mental health services, including those addressing substance use and somatic health, to optimize outcomes. Moreover, the substantial number of patients experiencing suicidal ideation during rehabilitation, primarily among patients with current MHDs, highlights the need for a proactive approach to suicide prevention, especially during the critical period around discharge.

A biopsychosocial approach is essential to understanding the complex interplay between psychological factors and the consequence of a physical injury or illness. If we ignore patients`mental health history, particularly their status at the time of injury or illness, we may overestimate the impact of the injury or illness on mental health, when in reality causality is more intertwined. This reinfoces the importance of identifying previous and current MHDs, including SUDs, at an early stage of rehabilitation to enable targeted interventions and to ensure tailored discharge processes.
